# Vacuolization, Dilatation, Hyaline Cast, Debris or Degeneration: Which One Is the Most Correlated Item to Score the Kidney Damage Pathologically in Cisplatin Induced Nephrotoxicity Model?

**DOI:** 10.5812/numonthly.8623

**Published:** 2013-08-04

**Authors:** Farzaneh Ashrafi, Mehdi Nematbakhsh, Hamid Nasri, Ardeshir Talebi, Sayed Mohsen Hosseini, Mehdi Ashrafi

**Affiliations:** 1 Water and Electrolytes Research Center, Department of Physiology, Isfahan University of Medical Sciences, Isfahan, IR Iran; 2 Department of Internal Medicine, Isfahan University of Medical Sciences, Isfahan, IR Iran; 3 Isfahan^MN^ Institute of Basic and Applied Sciences Research, Isfahan, IR Iran; 4 Department of Clinical Pathology, Isfahan University of Medical Sciences, Isfahan, IR Iran; 5 Depatrment of Industrial Engineering, Tarbiat Moddaress University, Tehran, IR Iran

**Keywords:** Dilatation, Hyaline, Degeneration, Kidney Failure, Chronic

## Abstract

**Background:**

Nephrotoxicity is characterized and scored by many parameters such as vacuolization, dilatation, hyaline cast, debris or degeneration in injured renal tissue. In this short report, we attempt to find, the most correlated parameters with kidney tissue pathology damage score (PDS) in Cisplatin-induced nephrotoxicity.

**Method:**

A total of 207 normal and toxic rats’ kidney tissue (induced by Cisplatin) were evaluated for toxicity intensity by two methods. In the first method, the tissue damage was scored from 0 to 4, and in the second method the percentage of vacuolization, dilatation, hyaline cast, debris or degeneration were determined. The data was analyzed using stepwise discriminant function and regression analysis.

**Results:**

The variables having the higher discriminant function coefficient were hyaline cast, dilatation, and degeneration. The linear regression model and the prediction function to determine the kidney tissue PDS were generated as below.

PDS = 0.445 + 0.035 × hyaline cast + 0.013 × dilatation + 0.020 × degeneration

**Conclusion:**

According to this finding it is suggested that presence of hyaline cast and dilatation, and then degeneration in the sample of toxic renal tissue are the most important item to score the damage intensity.

## 1. Introduction

Cisplatin (cis-diamminedichloroplatinum (II), CDDP) is an antineoplastic drug used in the treatment of many solid-organ cancers. Its major side effect is nephrotoxicity; 20% of patients receiving high-dose CDDP have severe renal dysfunction (1). CDDP nephrotoxicity primarily causes tubulo-interstitial lesions. In animal models CDDP damages the proximal tubules, specifically the S3 segment of the outer medulla of kidney. The glomerulus has no obvious morphologic changes (2, 3).

Biopsies obtained 3 to 60 days after dosing revealed segmental degeneration, necrosis, and desquamation of the epithelial cells in the pars convoluta and pars recta of the proximal tubules and the distal tubules (1). In patients with acute renal failure, the major lesion is acute necrosis mostly in proximal convoluted tubules (4).

Pathological evaluation of renal tissue is a well-known method for estimation of renal injury in experimental models of CDDP nephrotoxicity. Multiple parameters such as vacuolization, dilatation, hyaline cast, debris or degeneration, ect. should be inspected by nephropathologist in the evaluation process of injured renal tissue, but it is not clear which one is the most important item for scoring the intensity of renal injury in the nephrotoxicity model.

Our purpose in this study is to find out the significant morphologic variables in prediction of renal injury in rat models of nephrotoxicity induced by CDDP.

## 2. Methods

During the past three years, our team was involved in series of different basic researches to evaluate the CDDP induced nephrotoxicity in rats’ model. In general, our researches were performed in three groups of animals. The first group was assigned as the negative control group which received placebo (saline) during the study. The second group was assigned as the positive control group which treated with a single dose of CDDP (6-7 mg/kg, ip) and sacrificed one week later. The third group was assigned as experimental group which treated with a single dose of CDDP plus a supplementation agent such as antioxidants, and sacrificed one week later. In this report we are not attempt to evaluate the effect of supplementation agents on biochemistry parameters or tissue damages, instead we considered the damage intensity alone and independently in the kidneys. Accordingly, a total of 207 normal and abnormal kidney tissues were fixed in 10% neutral formalin solution and were embedded in paraffin for histopathological staining. Hematoxylin and Eosin stain was applied to examine the damage. All tissue samples were evaluated by two expert nephropathologists. The first pathologist was asked to grade the toxicity intensity from 0 to 4. The score of zero stands for normal tissue, and the score of 4 corresponds to more than 80% of tissue damage. The second nephropathologist was asked to report the percent of vacuolization, dilatation, hyaline cast, debris or degeneration (called pathology parameters) in each sample. Both nephropathologists were blind to the study.

### 2.1. Statistical Analysis

Data are reported as mean ± SEM. The pathology parameters and pathology damage score (PDS) were subjected to correlation analysis using Spearman test. To find the best prediction function, all the predictor variables (pathology parameters) were subjected to stepwise discriminant function analysis, the statistical significance was assessed using Wilks’ lambda. The variables having the higher discriminant function co-efficient were included in the discriminant function for developing the formula. Significant variables in discriminant analysis enter in the linear regression model; the significant of the coefficient for each variable were determined and the prediction function was generated.

## 3. Results

**Figure 1.**
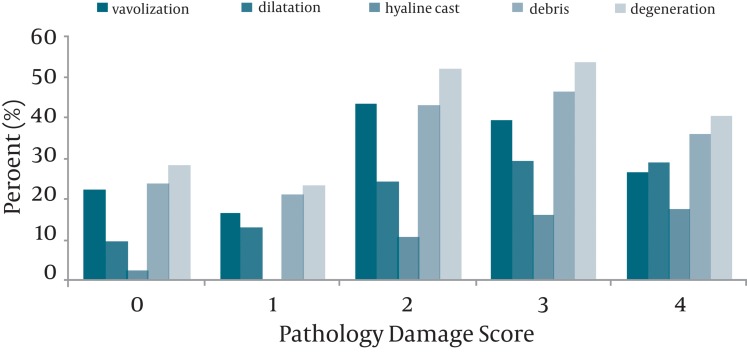
The Percent of Pathological Parameters for Each Pathological Damage Score

The percent of pathological parameters correspond to each PDS just as descriptive data is shown in Figure 1. The Pearson correlation coefficient (r) for the correlation between each pathological parameter (vacuolization, dilatation, hyaline cast, debris or degeneration) and PDS was tabulated in Table 1. According to data analysis mentioned in the statistical analysis, the variables having the higher discriminant function coefficient were hyaline cast, dilatation, and degeneration. These significant variables in discriminant analysis enter in the linear regression model and the prediction function was generated as shown below.

PDS = 0.445 + 0.035 × hyaline cast + 0.013 × dilatation + 0.020 × degeneration r = 0.60

**Table 1.** The Pearson Correlation Coefficient (r) for the Correlation Between Each Pathological Parameter and Pathology Damage Score. The Hyaline Cast and Dilatation Were More Correlated With the Pathology Damage Score than Other Parameters.

Pathological Parameters	Vacuolization	Dilatation	Hyaline Cast	Debris	Degeneration
**Pathology damage score**	0.231	0.461	0.632	0.331	0.313
**P value**	< 0.0001	< 0.0001	< 0.0001	< 0.0001	< 0.0001

On the other hand, and as indicated in Table 1, the hyaline cast and dilatation were more correlated with PDS than other parameters. The stepwise regression analysis also indicated that the regression coefficients for the hyaline cast and dilatation were statistically significant (P < 0.001), and based on these two variables to predict the PDS from the pathology parameters, the best prediction line is as following:

PDS = 0.935 + 0.057 × hyaline cast + 0.018 × dilatation r = 0.60

## 4. Discussion

CDDP nephrotoxicity causes tubular lesions (1). It damages the proximal tubules, specifically the S3 segment in the outer medulla (1, 5). In general, the glomerulus has no obvious morphologic changes (3). Morphological changes in kidney after CDDP treatment had been reported in several studies. In one report from Ravindra marked dilation of proximal convoluted tubules with slogging of the almost entire epithelium due to desquamation of the tubular epithelium after CDDP treatment was evident. Cellular debris in the tubular lumen and increased tissue in the interstium were also seen after CDDP induced acute kidney injury (5).

In another investigation after CDDP treatment focal acute tubular necrosis primarily affecting the distal and collecting tubules causing dilation of convoluted tubules and formation of casts in autopsy samples had been reported (6).

According to our findings we suggest pathologists firstly to consider presence of hyaline cast and dilatation, and then degeneration in sample of kidney tissue toxicity induced by CDDP. Indeed, hyaline casts and tubular dilatation, revealed more progressed lesions of the tubular cell injury (7-9).
